# A Watershed Year for an Update on the Genetics of Alcoholism

**Published:** 2012

**Authors:** Robert W. Williams, Antonio Noronha

**Affiliations:** **Robert W. Williams, Ph.D.,***Center for Integrative and Translational Genomics, The University of Tennessee Health Science Center, Memphis, Tennessee.*; **Antonio Noronha, Ph.D.,***Division of Neuroscience and Behavior, National Institute on Alcohol Abuse and Alcoholism, Bethesda, Maryland.*

It is easy to think of genetics as the study of genes, but given our current knowledge of genetics, this definition is now considered inadequate. Genetics is the study of differences among individuals—even between identical twins. We know that some differences between individuals are linked to variations in DNA sequence (i.e., the genome), but most differences actually are caused by complex interactions between our genetic endowment and the many environments to which we are exposed, by choice and fate.

The genetics underlying the body’s responses to alcohol are a superb example of the complexity of the issues related to the study of genetics. The role of the environment—including familial and social setting, age, and exposure—is obvious. Yet a complementary role of genes was doubted for many decades, and it was not put on a firm scientific foundation until the 1970s and 1980s. This coincided roughly with the creation of the National Institute on Alcohol Abuse and Alcoholism (NIAAA) by Congressional act on New Year’s Eve, 1970.

From its inception, NIAAA has been at the vanguard of research in genetics using an array of powerful methods and resources. The 13 insightful reviews in this issue highlight the cutting edge of molecular and statistical genetic analysis.

Now, moving forward, we can consider 2012 a watershed year in genetics and genomics. Two short years ago, fewer than 10 humans had had their genomes fully sequenced. Today more than 10,000 individuals have been sequenced—a rate of increase that puts Moore’s Law^1^ to shame. Recent advances in sequencing technology have broken through the cost barrier. Ten years from now, many of us will have been sequenced and even resequenced multiple times as part of our routine medical record.

It is easy to overpromise solutions, but we can be optimistic that finding effective cures to diseases such as alcoholism will not be limited by technology. The next challenge will be to determine how best to use this technology to devise rational and individualized approaches to prevent, intervene in, and cure complex disorders such as alcohol abuse and alcoholism. Read the great reviews here to see how far we have come in the last decade, and definitely stay tuned for the next decade!

## Footnotes

1 Moore’s Law predicts the doubling of the number of transistors on a computer chip every 18 months to 2 years. It is based on an observation made in 1965 by Gordon Moore, Intel co-founder, about how the number of transistors in integrated circuits had increased since the invention of that device and his suggestion that the increase would continue into the future.

